# Higher Seed Rates Enlarge the Effects of Wide-Belt Sowing on Root Length Density, Thereby Improving Nitrogen Uptake and Use Efficiencies in Winter Wheat

**DOI:** 10.3390/plants13172476

**Published:** 2024-09-04

**Authors:** Yuechao Wang, Wen Li, Yaoyao Deng, Jianfu Xue, Zhiqiang Gao

**Affiliations:** College of Agriculture, Shanxi Agricultural University, Taigu, Jinzhong 030801, China; wychao0714@163.com (Y.W.); liwen001216@163.com (W.L.); yydeng567@163.com (Y.D.); gaosxau@163.com (Z.G.)

**Keywords:** wide-belt sowing, seed rate, root length density, nitrogen uptake, nitrogen use efficiency

## Abstract

The optimized sowing method and appropriate seed rate can improve wheat N use efficiency. However, the interactive effect of the sowing method and seed rate on N use efficiency, particularly N uptake and root length density, are unclear. A field experiment was conducted for two growing seasons in southern Shanxi province, China, using a split-plot design with the sowing method as the main plot (wide-belt sowing, WBS, and conventional narrow-drill sowing, NDS) and seed rate as the sub-plot (100–700 m^−2^). Our results showed that WBS had a significant and positive effect on N use efficiency (yield per unit of available N from the fertilizer and soil, by 4.7–15.4%), and the relatively higher seed rates (>300 or 400 m^−2^) enlarged the effects. The N use efficiency increases under WBS were mainly attributed to the increases in N uptake before anthesis, resulting from the promoted nodal roots per plant and per unit area, and root length density in the top layer(s). WBS promoted N translocation and the N harvest index, resulting in equivalent grain protein concentration and processing quality compared to NDS. Thus, adopting higher seed rates (>300 m^−2^) combined with WBS is recommended for achieving greater N efficiencies while maintaining the grain protein concentration and processing quality of winter wheat.

## 1. Introduction

In northern China, wide-belt sowing (WBS) has been proven to significantly increase not only yields but also nitrogen use efficiency (NUE) compared with traditional narrow-drill sowing (NDS) [1]. N is the most important nutrient determining wheat (*Triticum aestivum* L.) grain yield and quality. NUE reflects the relationship between crop yield and the quantity of applied N or available N in the soil [2,3,4]. Enhancing NUE is crucial for increasing wheat yield and improving grain quality. It is necessary to enhance both the crop’s ability to absorb N (N uptake efficiency, NUPE) and the ability to utilize absorbed N for grain production (N utilization efficiency, NUTE) [5,6,7]. NUPE is the ratio of the total above-ground N at maturity to the N supply (the sum of soil N supply and fertilizer N application). NUTE is the grain yield produced per unit area of above-ground N, reflecting the capacity of using absorbed N for grain production.

NUPE and NUTE are influenced by the sowing method and seed rate (i.e., planting density). It was found that WBS primarily improved NUE by enhancing NUPE, thereby increasing yield [8,9,10]. Liu et al. [11] and Wang et al. [1] stated that the increased N uptake before and after anthesis under WBS led to N uptake and yield increase of over 10% at maturity. Roots are the primary organs for wheat to absorb water and nutrients, and the number and length density of roots largely determine N uptake and yield performance [12,13,14]. Zheng et al. showed that the number of roots per wheat plant under WBS was 8.4% to 18.5% higher than NDS, with root length density and root surface area density more than 25% and 22% higher, respectively, compared with conventional row sowing [15]. Probably because the distance between individual plants within a row under WBS is enlarged relative to NDS, the allocation of more below- and above-ground spaces to individuals increases access to nutrients and produces more tillers [16,17]. Other studies have shown that WBS increased wheat yield and N uptake, but without significant impact on grain protein concentration or processing quality [11,18]. The equivalent increased percentages in grain nitrogen accumulation and yield led to an unchanged grain protein concentration.

In addition to the sowing method, seed rate also significantly affects wheat N uptake, utilization, dry matter production, and yield formation, as well as NUE. Dong et al. showed that by increasing the seed rate from 135 m^−2^ to 405 m^−2^, above-ground N and yield significantly increased [19]. Arduini et al. also showed that a higher seed rate resulted in a higher N uptake in durum wheat [20]. However, other studies [10,21,22] found that as the seed rate increased, yield and N uptake increased first and then decreased, with the highest yield and the highest above-ground N at 405–410 m^−2^. The number of roots, root length density, and root vitality are important indicators of wheat root absorption capacity [6]. Gaju et al. stated that a greater root length density was beneficial for absorbing more N [23]. Dai et al. [22] and Dong et al. [19] found that as the seed rate increased, the increased number of nodal roots per unit area and root length density mainly explained the increases in above-ground N and yield. The seed rate was found to affect wheat N uptake and yield formation significantly and also impact grain protein concentration and processing quality, but the extent of the impact varied greatly among studies. Tompkins et al. [24] and Dong et al. [25] showed that grain protein concentration tended to increase with seed rate. Geleta et al. [26] concluded that protein content decreased with an increased seeding rate of up to 130 kg ha^−1^. However, Carr et al. [27] and Ozturk et al. [28] indicated there was no significant difference in grain protein concentration among seed rates. These discrepancies might be caused by the different genotypes of the wheat varieties used, climatic conditions, management practices, and yield levels [29]. For instance, Zhang et al. showed that with 240 kg ha^−1^ N application, grain protein concentration increased as the seed rate increased, but without N application, the opposite situation was observed [30].

Our previous studies showed that both the sowing method and seed rate significantly affected yield, with a significant interaction effect [31]. However, so far, there is a lack of comprehensive reports on the effects of the sowing method and seed rate, especially their interaction, on N uptake, utilization, and grain quality. In the present study, N uptake and utilization, root number and length density, grain protein concentration, and processing quality were investigated for winter wheat in a two-season field experiment with two sowing methods (NDS and WBS) and seven seed rates (100 to 700 m^−2^, with an interval of 100 m^−2^). The objectives of this study were as follows: (1) to determine the interactive effect of the sowing method by seed rate on N uptake, utilization and use efficiencies, root characteristics, and grain quality and (2) to identify the relationship between increases in nitrogen uptake and root number and length density by WBS. We hypothesize that WBS can increase root number and length density, thereby promoting N uptake and NUE, but the effect degree may differ among seed rates.

## 2. Results

### 2.1. N Use, Uptake, and Utilization Efficiencies

**Figure 1:**
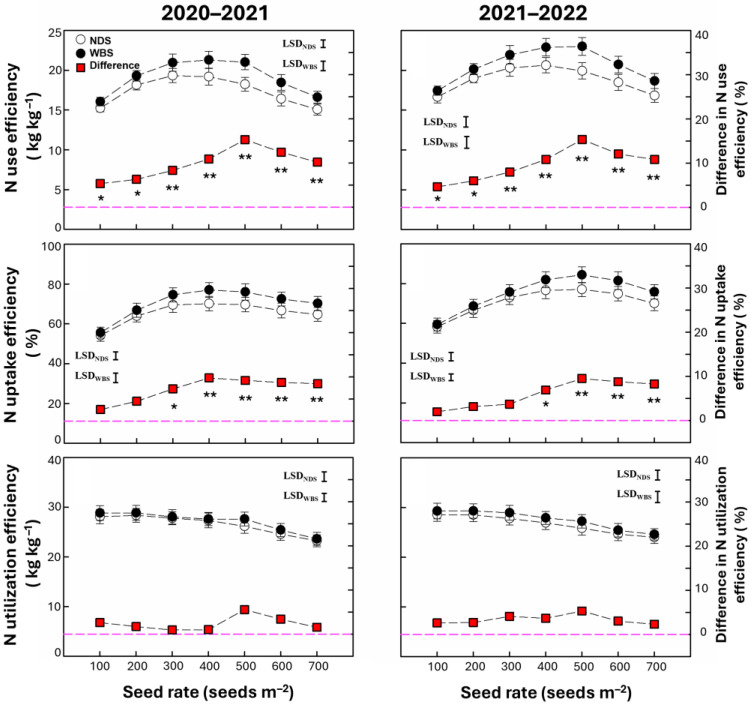
N use efficiency, uptake efficiency, and utilization efficiency of winter wheat with narrow-drill sowing (NDS) and wide-belt sowing (WBS) in two growing seasons. Data are means and error bars are SEs (n = 4). The dashed line is a reference line indicating that the difference in the percentage of efficiency between WBS and NDS is zero. * and ** indicate that there is a significant difference between WBS and NDS at the specific seed rate, according to Student’s *t*-test at α = 0.05 and 0.01, respectively.

N use efficiency and uptake efficiency were significantly affected by the sowing method, seed rate, and their interaction, but N utilization efficiency was only significantly affected by the seed rate (Table 1). Both N use efficiency and N uptake efficiency increased initially and then decreased as the seed rate increased in both seasons (Figure 1). N use efficiency was 5.4–15.3% and 4.7–15.4% higher under WBS compared with NDS in 2020–2021 and 2021–2022, respectively. The highest increase in N use efficiency was obtained at SR500 in both seasons. N uptake efficiency was 7.3–9.8% and 6.9–9.5% higher under WBS compared with NDS at seed rates of 300–700 m^−2^ in 2020–2021 and 400–700 m^−2^ in 2021–2022, respectively. The highest increase in N uptake efficiency was obtained at SR400 and SR500 in 2020–2021 and 2021–2022, respectively. N utilization efficiency tended to decrease as the seed rate increased under both sowing methods, and no difference was observed between WBS and NDS at any seed rate in either season.

### 2.2. N Uptake at Anthesis and Maturity

**Figure 2:**
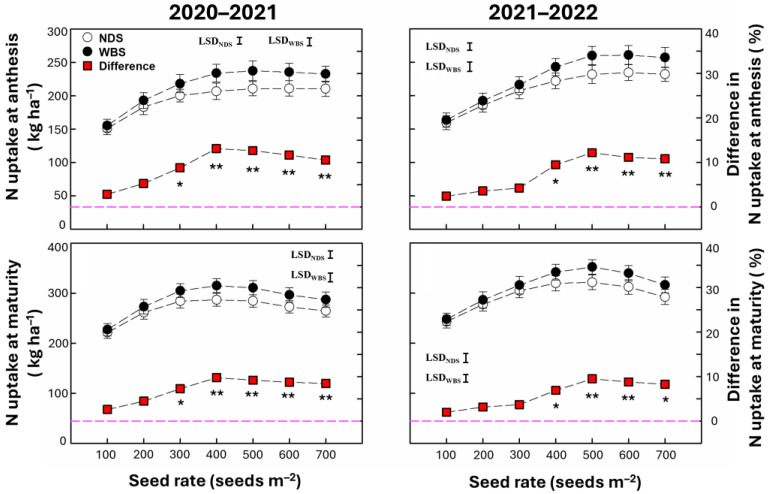
N uptake at anthesis and maturity of winter wheat with narrow-drill sowing (NDS) and wide-belt sowing (WBS) in the 2020–2021 and 2021–2022 growing seasons. Data are means and error bars are SEs (n = 4). The dashed line is a reference line indicating that the difference in the percentage between WBS and NDS is zero. * and ** indicate that there is a significant difference between WBS and NDS at the specific seed rate, according to Student’s *t*-test at α = 0.05 and 0.01, respectively.

The N uptake at anthesis and maturity was significantly affected by the sowing method, seed rate, and their interaction (Table 1). The N uptake at anthesis increased initially and then stabilized as the seed rate increased under both sowing methods in both seasons (Figure 2). In the 2020–2021 season, the N uptake at anthesis was 8.8–13.1% higher under WBS compared with NDS, except at the seed rates of 100 and 200 m^−2^. In the 2021–2022 season, the N uptake at anthesis was 9.5–12.2% higher under WBS at 400–700 m^−2^ compared with NDS. The N uptake at maturity increased initially and then decreased as the seed rate increased in both seasons. The N uptake at maturity was 7.3–9.8% and 6.9–9.5% higher under WBS at 300–700 m^−2^ in 2020–2021 and 400–700 m^−2^ in 2021–2022, respectively. The highest increases in the N uptake at anthesis and maturity were obtained at SR400 in 2020–2021 and at SR500 in 2021–2022.

### 2.3. Root Length Density

**Figure 3:**
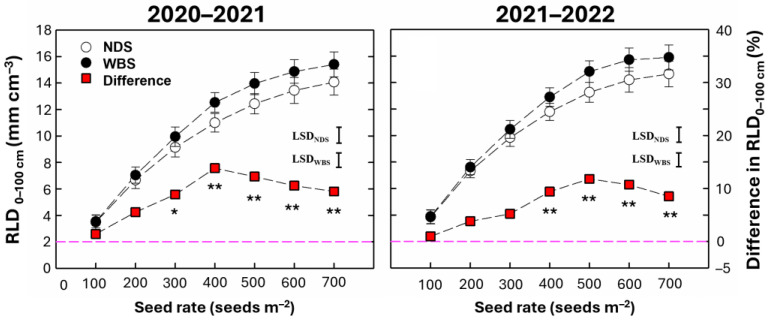
Root length density in the 0–100 cm soil layer (RLD_0–100_) in the 2020–2021 and 2021–2022 growing seasons. Data are means and error bars are SEs (n = 4). The dashed line is a reference line indicating that the difference percentage between WBS and NDS is zero. * and ** indicate that there is a significant difference between WBS and NDS at the specific seed rate, according to Student’s *t*-test at α = 0.05 and 0.01, respectively.

The RLD in the soil at a depth of 0–100 cm was significantly affected by the sowing method, seed rate, and their interaction (Table 1). As the seed rate increased, the RLD tended to continuously increase under both sowing methods in both seasons (Figure 3). Significant increases by WBS compared with NDS were 9.0–13.9% and 8.5–11.8% at seed rates of 300–700 m^−2^ in 2020–2021 and 400–700 m^−2^ in 2021–2022, respectively. The highest increases occurred at SR400 and SR500 in 2020–2021 and 2021–2022, respectively.

**Figure 4:**
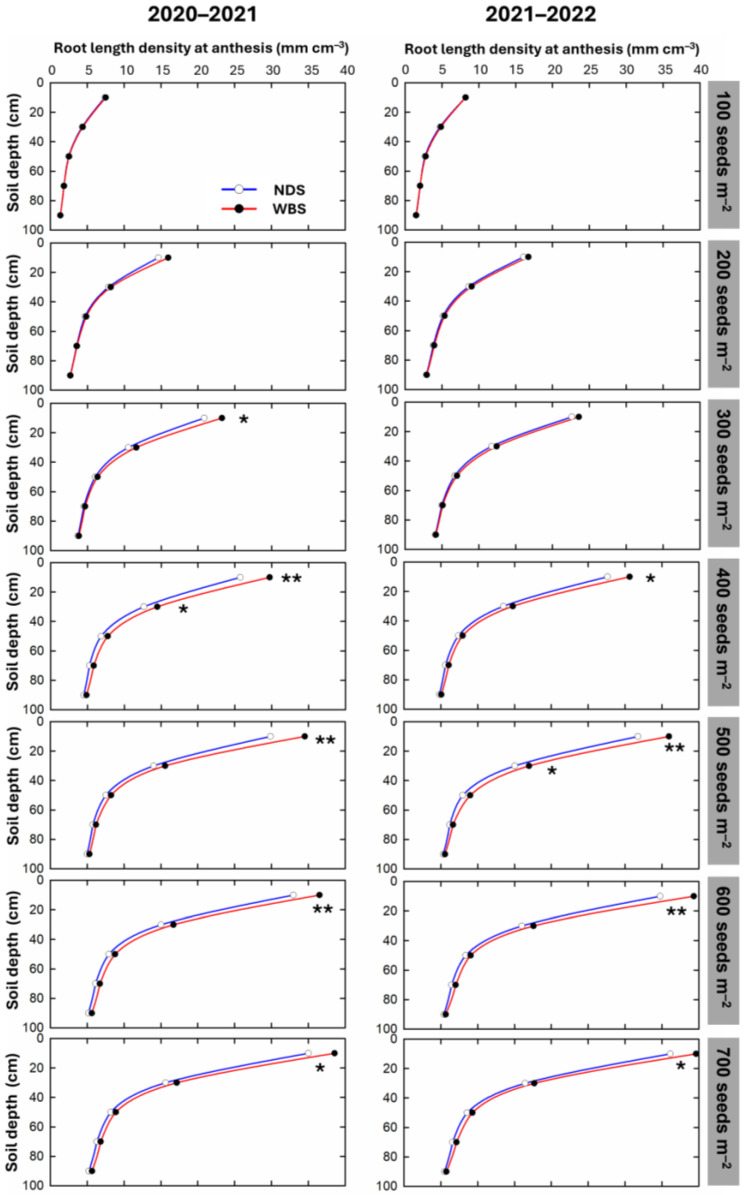
Root length density at anthesis at the 0–100 cm soil depth in the 2020–2021 and 2021–2022 growing seasons. * and ** indicate there is a significant difference between narrow-drill sowing (NDS) and wide-belt sowing (WBS) at the specific seed rate and soil layer, according to Student’s *t*-test at α = 0.05 and 0.01, respectively.

As the seed rate increased, the RLD at all soil depths gradually increased under both sowing methods in both seasons (Figure 4). In 2020–2021, no significant difference in the RLD between WBS and NDS was observed in any soil layer at a seed rate of either 100 or 200 m^−2^. However, significant increases of 10.1–15.6% were observed in the top soil layer at a depth of 0–20 cm at seed rates of 300–700 m^−2^ under WBS. In addition, a significant increase of 14.4% in the RLD in the soil layer at a depth of 20–40 cm was observed at a seed rate of SR400. In 2021–2022, there was no significant difference in RLD in any soil layer at any seed rate of 100–300 m^−2^. Significant increases of 9.7–13.2% were observed in the top soil layer at a depth of 0–20 cm at seed rates of 400–700 m^−2^. Furthermore, a significant increase of 12.9% by WBS in the RLD occurred in the soil layer at a depth of 20–40 cm at SR500.

### 2.4. Nodal Roots per Plant and Unit Area

**Figure 5:**
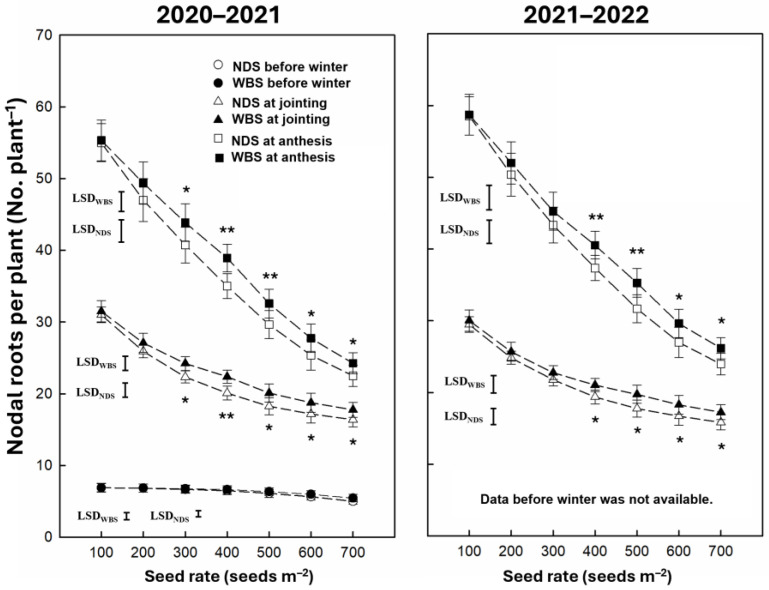
The nodal roots per plant before winter at jointing and anthesis of winter wheat with narrow-drill sowing (NDS) and wide-belt sowing (WBS) in two growing seasons. Data are means and error bars are SEs (n = 4). * and ** indicate there is a significant difference between WBS and NDS at the specific seed rate, according to Student’s *t*-test at α = 0.05 and 0.01, respectively.

The nodal roots per plant at jointing and anthesis were significantly affected by the sowing method, seed rate, and their interaction, but the nodal roots per plant before winter were significantly affected only by the seed rate (Table 1). The nodal roots per plant at all measuring stages decreased with an increasing seed rate under both sowing methods in both seasons (Figure 5). In 2020–2021, no significant difference in the nodal roots per plant at jointing and anthesis was observed at a seed rate of either 100 or 200 m^−2^. Significant increases of 8.4–11.3% and 7.7–11.3% were observed at seed rates of 300–700 m^−2^ at jointing and anthesis, respectively. In 2021–2022, there was no significant difference in the nodal roots per plant at jointing and anthesis at a seed rate of 100–300 m^−2^. Significant increases of 8.5–11.2% and 8.5–11.4% were observed at seed rates of 400–700 m^−2^ at jointing and anthesis, respectively.

**Figure 6:**
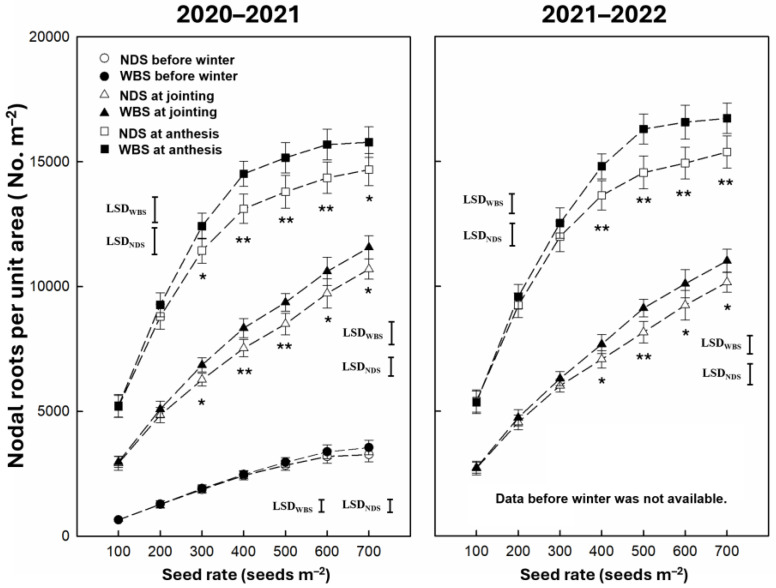
The nodal roots per unit area before winter at jointing and anthesis of winter wheat with narrow-drill sowing (NDS) and wide-belt sowing (WBS) in two growing seasons. Data are means and error bars are SEs (n = 4). * and ** indicate there is a significant difference between WBS and NDS at the specific seed rate, according to Student’s *t*-test at α = 0.05 and 0.01, respectively.

The nodal roots per unit area at jointing and anthesis were significantly affected by the sowing method, seed rate, and their interaction, but the nodal roots per unit area before winter were significantly affected only by the seed rate (Table 1). The nodal roots per unit area at all measuring stages increased with an increasing seed rate (Figure 6). In 2020–2021, the nodal roots unit per area were 8.1–10.7% and 7.5–10.7% higher under WBS than NDS at jointing and anthesis, respectively. In 2021–2022, there were significant increases by WBS of 8.4–11.8% and 8.5–12.0% at jointing and anthesis, respectively.

### 2.5. Grain N Accumulation Related Traits

N translocation, grain N accumulation, their ratio (NT/GN), and the N harvest index were significantly affected by the sowing method, seed rate, and their interaction, and only the seed rate affected post-anthesis N uptake of winter wheat (Table 2). Grain N accumulation and N translocation increased and then decreased with an increasing seed rate under both sowing methods and in both seasons. Furthermore, the difference in grain N accumulation between sowing methods increased from 7.8–8.0% at SR200, peaked at 13.9–14.5% at SR500, and then decreased to 8.8–9.7% at SR700. The difference in N translocation performed similarly to grain N accumulation, and the corresponding values were 12.4–14.2%, 23.3–24.5%, and 12.7–15.2%. Post-anthesis N uptake increased first and then decreased with an increasing seed rate, and there was no significant difference between NDS and WBS at any seed rate and in either season. NT/GN increased with an increasing seed rate, and NT/GN under WBS significantly increased by 6.0–8.8% at a seed rate of 400–600 m^−2^ than under NDS in 2020–2021, and by 8.3–9.0% at seed rates of 400 and 500 m^−2^ in 2021–2022. Averaged across all seed rates, WBS increased the N harvest index by 2.33% (*p* < 0.05) and 3.42% (*p* < 0.05) in 2020–2021 in 2021–2022, respectively. However, the effects of WBS on the N harvest index were inconsistent across seed rates and seasons.

### 2.6. Grain Protein Concentration, Yield, and Processing Quality-Related Traits

Only the seed rate affected the grain protein concentration, water absorption ratio, wet gluten content, and dough stability time; however, the grain protein yield was significantly affected by the sowing method, seed rate, and their interaction (Table 3). The grain protein concentration, water absorption ratio, wet gluten content, and dough stability time increased with an increasing seed rate. The grain protein yield significantly increased and then decreased with an increasing seed rate under both sowing methods. Furthermore, the grain protein yield under WBS increased by 6.2–14.5% and 6.2–13.9% in 2020–2021 and 2021–2022, respectively. The highest increase in the grain protein yield was obtained at SR500 in both seasons.

### 2.7. Correlation Analysis

**Figure 7:**
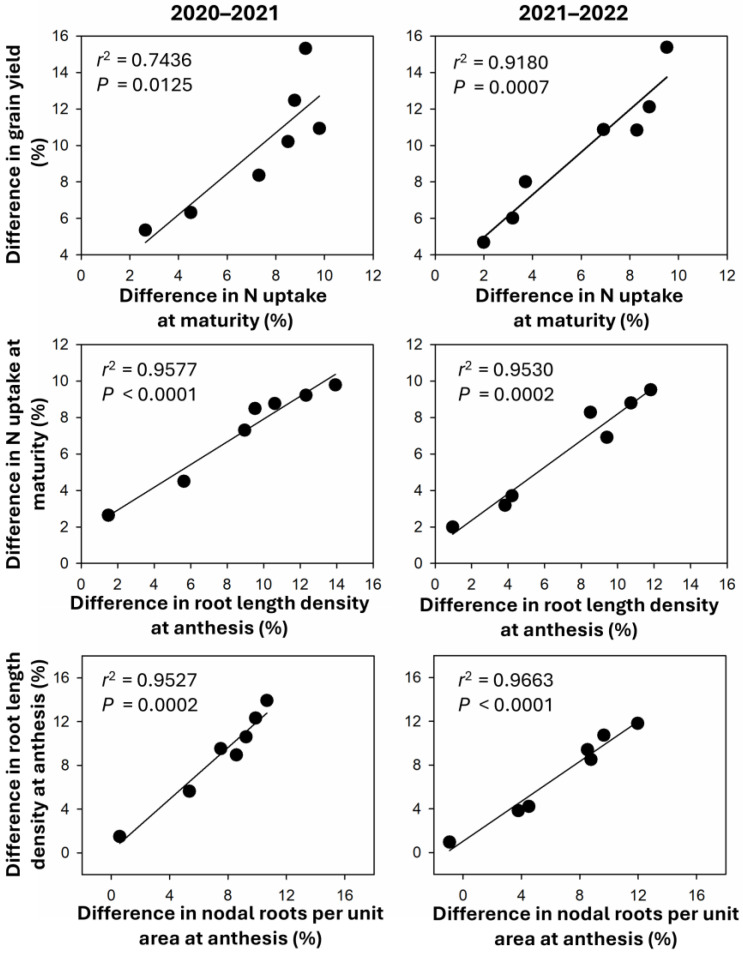
The relationship between differences in yield, N uptake, and root traits.

In both seasons, the differences in grain yield between WBS and NDS were significantly and positively correlated with the difference in N uptake at maturity (Figure 7). The difference in N uptake at maturity was significantly and positively correlated with the difference in root length density in the soil at a depth of 0–100 cm at anthesis. A significant and positive linear relationship between differences in root length density in the soil at a depth of 0–100 cm and nodal roots per unit area at anthesis was observed in both seasons.

## 3. Discussion

Previous research demonstrated that higher seed rates amplify the effects of WBS on canopy radiation capture, distribution, and use efficiency, ultimately enhancing crop yield [31]. In this study, we found that WBS also improved NUE. Several studies similarly reported that WBS promoted nitrogen uptake, thereby enhancing NUE [1,8,9,10]. However, the reasons for increased NUE differed across various seed rates. When the seed rate was 200 or 300 m^2^ or less, the NUE improvement under WBS was attributed to relatively modest increases in both nitrogen uptake efficiency (NUPE) and nitrogen utilization efficiency (NUTE) (*p* > 0.05). In contrast, at seed rates exceeding 200 or 300 m^2^, the increase in NUPE (*p* < 0.05) primarily contributed to the enhancement in NUE. These findings suggested that the increase in NUPE, rather than NUTE, was responsible for the enhanced NUE observed with WBS, particularly at relatively higher seed rates (400–500 m^2^).

The NUPE reflects the capacity of plants to uptake available N, which comes from applied fertilizer and soil [5,21]. In our study, N uptake was used as a proxy for NUPE since the experiment did not involve variations in N management (i.e., available N was consistent across all treatments). At all seed rates, WBS did not significantly affect N uptake after anthesis. However, significant increases of 8.8–13.1% in N uptake at anthesis were observed at seed rates above 200 or 300 m^2^, leading to increases of 6.9–9.8% in N uptake at maturity. Correlation analysis indicated that the increase in N uptake due to WBS could account for at least 74.4% of the variation in the increase in grain yield (Figure 7). Our previous study showed that WBS significantly increased N uptake at maturity by 10–12% because of a balanced increase in N uptake both before and after anthesis [1]. Liu et al. [11] also found that WBS increased N uptake at maturity by 10%, although the contributions of pre- and post-anthesis nitrogen uptake varied among bread wheat varieties and seasons. These findings collectively suggested that WBS enhanced wheat plants’ capacities to uptake more N, thereby increasing yield. However, the extent of this enhancement, particularly after anthesis, may depend on genotype and climatic conditions, warranting further investigation.

A key finding of this study was that WBS significantly affected root length density within the soil at a depth of 0–100 cm (RLD_0–100_). At seed rates of 200 or 300 m^−2^ or less, WBS did not significantly affect root length density at anthesis. However, significant increases of 8.5–13.9% in RLD_0–100_ were observed at seed rates above 200 or 300 m^−2^ (Figure 3), primarily because of increased root length density in the top 0–20 cm soil layer (Figure 4). Notably, significant increases in the 20–40 cm soil layer also contributed to the greatest overall increase in RLD_0–100_ at seed rates of 400 or 500 m^−2^. Zheng et al. [15] similarly reported that WBS increased root length density within the soil at a depth of 0–120 cm, with the most significant contributions coming from the 0–40 cm soil layer. Root architecture, including root length density and the spatial distribution of roots within the soil, determines the capacity of a crop to uptake nutrients from the soil [14,22,32]. Correlation analysis indicated that the increase in RLD_0–100_ due to WBS could explain at least 95.3% of the variation in the increase in N uptake. These results suggested that WBS enhanced N uptake by increasing the root length density in the upper soil layers, particularly at relatively higher seed rates (>300 or 400 m^−2^). Dai et al. [22] reported that nodal roots play a crucial role in determining root length density. Consistent with RLD_0–100_, nodal roots per plant and per unit area were significantly promoted by WBS at relatively higher seed rates (>300 or 400 m^−2^), with the greatest promotions observed at SR400 or SR500 at jointing and anthesis. The increase in root length density due to WBS was significantly and positively related to the increase in nodal roots per unit area at anthesis (*r*^2^ > 0.95, *p* < 0.01). In this study, since the ratio of plants to seeds was consistent between sowing methods, plant density was the same for both NDS and WBS [31]. Therefore, the nodal roots per plant solely determined the nodal roots per unit area. In summary, WBS increased nodal roots per plant compared with NDS, resulting in increased nodal roots per unit area, root length density, nitrogen uptake, and nitrogen use efficiency, ultimately enhancing grain yield. Moreover, the relatively higher seed rates (>300 or 400 m^−2^) magnified the effects of WBS.

Wheat grain protein is a critical component of the global human diet. While increasing wheat yield, it is essential to maintain and improve grain quality, including protein concentration and flour processing quality [33]. However, wheat grain yield typically exhibits an inverse relationship with grain protein concentration, especially under conditions of insufficient N supply [34,35]. In this study, no significant effect of WBS was observed on the grain protein concentration, water absorption ratio, wet gluten content, or dough stability time in either season, consistent with the findings of Wu et al. [36], Zhao et al. [37], and Liu et al. [11]. In our published work, WBS increased grain yield by 4.7–15.4% [31], which was higher than the increase in N uptake (2.0–9.8%) observed in the present study. These results indicated that the increase in N acquisition did not keep pace with yield enhancement, generally leading to a reduction in grain N (protein) concentration. Importantly, the significant increase in the N harvest index, which reflects the efficiency of partitioning above-ground nitrogen uptake into the grain, compensated for this gap, resulting in stable grain protein concentration and thereby maintaining processing quality. N accumulation in grain at maturity typically originated from the following sources: N absorbed from the soil via root after anthesis (NU_post_) and N remobilized or translocated from vegetative tissues (NT) [23,35,38,39]. No significant increase in NU_post_ was observed in either season; thus, the increase in NT played a vital role in enhancing the nitrogen harvest index and maintaining grain protein concentration and processing quality. The increase in NT due to WBS was also observed in a previous study using four strong-gluten wheat varieties [11].

## 4. Materials and Methods

### 4.1. Site Description, Experimental Design, and Crop Management

Field experiments were conducted at Gucheng Village (35°43′ N, 111°44′ E), Tangxing Town, Yicheng County, Shanxi Province, in two winter wheat growing seasons during 2020–2021 and 2021–2022. The soil type was classified as silty clay loam according to the USDA Soil Taxonomy, with a pH of 8.33–8.41, organic matter content of 19.52–20.38 g kg^−1^, total N content of 0.94–1.01 g kg^−1^, alkaline N content of 48.25–50.07 mg kg^−1^, Olsen P content of 19.23–19.55 mg kg^−1^, and available K content of 171.87–174.24 mg kg^−1^ in 0–20 cm soil. For 20–40 cm soil, the pH value was 8.36–8.46, organic matter content was 16.14–16.67 g kg^−1^, total N content was 0.81–0.92 g kg^−1^, alkaline N content was 37.36–40.11 mg kg^−1^, Olsen P content was 17.41–18.37 mg kg^−1^, and available K content was 161.44–169.17 mg kg^−1^. The mean bulk density in the 0–2 m soil profile was 1.31 g cm^−3^, and the mean field capacity was 24.1%.

**Figure 8:**
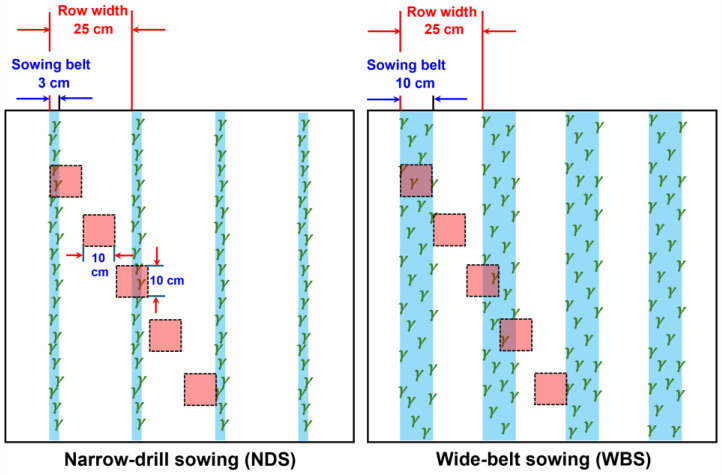
Sketch maps of the sowing methods and root sampling at anthesis.

This article describes the follow-up study of our previous report [31]. The treatments were laid out in a split-plot design with 4 replicates. The sowing method was designated as the main plot and the seed rate as the sub-plot. The sowing methods (Figure 8) comprised conventional NDS (sowing belt and row widths of 3 cm and 25 cm, respectively) and WBS (sowing belt and row widths of 10 cm and 25 cm, respectively), which are used widely for wheat production in the study region. The widely planted wheat cultivar Zhongmai175 was selected. For both sowing methods, the seven seed rates were 100, 200, 300, 400, 500, 600, and 700 m^−2^ (SR100, SR200, SR300, SR400, SR500, SR600, and SR700).

In order to control the sowing density more accurately, this experiment used manual sowing, and the plant density was counted at the three-leaf stage (Zadoks code 13). The rate of emergence did not significantly differ between the treatments in either year. N fertilizer was 225 kg N ha^−1^, with 135 kg N ha^−1^ as the base fertilizer and 90 kg N ha^−1^ as the topdressing during the jointing stage (Zadoks code 32), as described in Li et al. 2024. Before sowing, 150 kg P_2_O_5_ ha^−1^ and 90 kg K_2_O ha^−1^ were applied. The field was kept free of diseases and pests. Weeds were controlled by spraying with herbicide three times in each experimental season. Wheat crops were sowed on 15 October and 24 October in 2020 and 2021, respectively. In 2021, heavy rain in early October made the soil quite wet and not suitable for sowing, and thus, sowing was delayed for 9 days, compared with 2020.

Further details on this section were described in a previous study [31].

### 4.2. Sampling and Measurements

#### 4.2.1. Soil N Content before Sowing

Before basal fertilizer application, soil samples were collected from 0–20, 20–40, 40–60, 60–80, and 80–100 cm soil depths in the experimental field with 5 replicates using a five-spot-sampling method. The fresh soil samples were analyzed for total mineral N content (NO_3_^−^-N and NH_4_^+^-N) using a continuous flow analyzer (AutoAnalyzer 3, Bran+Luebbe, Norderstedt, Germany) according to the method described by Wagner in 1974 [40] and Benesch and Mangelsdorf in 1972 [41].

#### 4.2.2. N Uptake and Use Efficiencies

In the anthesis and maturity stages, wheat plants were sampled from a typical and central row over a length of 0.5 m (0.125 m^2^). The plant samples were separated into leaf bleads, stems (sheaths included), and ears at anthesis, and separated into straw (leaf blades, sheaths, and stems included) and ears. The ears were manually threshed and then divided into grain and grains, rachides, and glumes. The rachides and glumes were put together with straw. All parts were oven-dried to constant weight at 80 °C and then ground for analysis. The tissue N concentration (N mass per unit dry weight, mg g^−1^) was determined using an elemental analyzer (Rapid N Exceed, Elementar, Langenselbold, Germany). N uptake was calculated by multiplying the N concentration by dry weight.

Post-anthesis N uptake (NU_post_) was calculated as follows:

(1) $${NU}_{post}\left(kg\;{ha}^{-1}\right)=N\;uptake\;at\;maturity-N\;uptake\;at\;anthesis$$

The N translocation (NT) of accumulated above-ground N uptake before anthesis into developing grains was calculated as follows:

(2) $$NT\left(kg\;{ha}^{-1}\right)=Grain\;N\;at\;maturity-{NU}_{post}$$

The N harvest index (NHI) was calculated as follows:

(3) $$NHI\left(\%\right)=\frac{Grain\;N\;at\;maturity}{N\;uptake\;at\;maturity}\times 100$$

N use efficiency (NUE) was calculated as follows:

(4) $$NUE\left(kg\;{kg}^{-1}\right)=\frac{Grain\;yield}{N_{f}+N_{s}}$$

N uptake efficiency (NUPE) was calculated as follows:

(5) $$NUPE\left(\%\right)=\frac{N\;uptake\;at\;maturity}{N_{f}+N_{s}}\times 100$$

where N_f_ is the N fertilizer applied into the soil and N_s_ represents the total mineral N content (NO_3_^−^-N and NH_4_^+^-N) in the soil at a depth of 0–100 cm. The N_s_ values were 184.2 and 191.8 kg ha^−1^ before sowing in 2020 and 2021, respectively.

N utilization efficiency (NUPE) was calculated as follows:

(6) $$NUTE\left(kg\;{kg}^{-1}\right)=\frac{Grain\;yield}{N\;uptake\;at\;maturity}$$

#### 4.2.3. Grain Protein and Processing Quality

The grain protein concentration was calculated by multiplying the N concentration by a factor of 5.7 [25]. Grain protein yield was calculated by multiplying the grain protein concentration by grain yield. The wheat grains that were used to determine the wet gluten content, water absorption ratio, and dough stability time naturally were sun-dried and left in a dry and cool place for 3 months before being crushed into whole wheat flour. The wet gluten content was determined by using a gluten washing instrument (Perten, Alpnach, Switzerland) according to standards GB/T 5506.2-2008 [42]. The water absorption ratio and dough stability time were determined by using a FarinoGraph-E (Brabender, Duisburg, Germany) according to standard GB/T 14614-2019 [43].

#### 4.2.4. Nodal Root Number and Root Length Density

Before winter (Zadoks code 23), at jointing (Zadoks code 32) and anthesis (Zadoks code 65), wheat plants were dug out from a typical and central row over a length of 0.5 m (0.125 m^2^) and soaked in water. Then, the soil attached to the root zone were washed away and the nodal root number was recorded. Before winter in 2021, the plants were too small to record the nodal root number because of the delayed sowing date. The nodal roots per unit area (NRA) was calculated as follows:

(7) $$NRA\left(m^{-2}\right)=Nodal\;roots\;per\;plant\times plant\;density\;at\;three\;leaves\;stage$$

At anthesis, root samples were collected from a cross-section of a 10 cm × 10 cm square. In each sub-plot, five sampling sites were established (10 cm × 5, two row widths), as presented in Figure 8. We sampled the soil at a 10 cm depth, and samples of each 20 cm depth for all five sites were put together into a string mesh bag. For each bag, soil was washed away from the roots, and organic debris and other materials were divided from the wheat roots. The root length measurement was conducted using a flatbed scanner (V600 Photo Scanner, Epson, Los Alamitos, CA, USA) as described by Zheng et al. [15]. The root length density (RLD) at a specific soil layer was calculated as follows:

(8) $$RLD\left({mm\;cm}^{-3}\right)=\frac{Root\;length(mm)}{10\;cm\times 10\;cm\times 5\times 10\;cm\times 2}$$

### 4.3. Data Analysis

Statistical analyses were performed with Statistix 9.0 (Analytical Software, Tallahassee, FL, USA). Analysis of variance was conducted separately for each year. Differences in traits under NDS and WBS at the same seed rate were detected using Student’s *t*-test (*α* = 0.05). Pearson correlation analysis was conducted to determine the correlation coefficient (*r*). All graphical representations of the data were produced using SigmaPlot 12.5 (Systat Software Inc., Point Richmond, CA, USA).

## 5. Conclusions

Overall, WBS had a significant and positive effect on N use efficiency (4.7–15.4%), and the relatively higher seed rates (>300 or 400 m^−2^) enlarged the effects of WBS. The N use efficiency increase under WBS was mainly attributed to the increase in N uptake before anthesis. WBS increased nodal roots per plant compared with NDS, resulting in increased nodal roots per unit area and root length density, especially in the soil at a depth of 0–20 cm, thereby enhancing N uptake and use efficiencies. WBS promoted N translocation and enhanced the N harvest index, resulting in an equivalent grain protein concentration and processing quality compared with NDS. Thus, higher seed rates (>300 m^−2^) combined with WBS are recommended to achieve greater N efficiencies while maintaining the grain protein concentration and processing quality of winter wheat.

## Figures and Tables

**Table 1:** Analysis of variance for N uptake and use efficiencies, root length, and quantity-related traits.

Season	Source	NUE	NUPE	NUTE	NU_total_	NU_pre_	RLD	NRP_BW_	NRP_JS_	NRP_AS_	NRA_BW_	NRA_JS_	NRA_AS_
2020–	SM	**	**	ns	**	**	**	ns	**	**	ns	**	**
2021	SR	**	**	**	**	**	**	**	**	**	**	**	**
	SM × SR	**	**	ns	**	**	**	ns	**	**	ns	**	**
2021–	SM	**	**	ns	**	**	**	NA	**	**	NA	**	**
2022	SR	**	**	**	**	**	**	NA	**	**	NA	**	**
	SM × SR	**	**	ns	**	**	**	NA	**	**	NA	**	**

**, significant at the 0.01 probability level; ns, not significant at the 0.05 probability level; NA, not available; SM, sowing method; SR, seed rate; NUE, N use efficiency; NUPE, N uptake efficiency; NUTE, N utilization efficiency; NU_total_, N uptake at maturity; NU_pre_, pre-anthesis N uptake; RLD, root length density in the soil at a depth of 0–100 cm; NRP_BW_, nodal roots per plant before winter; NRP_JS_, nodal roots per plant at jointing stage; NRP_AS_, nodal roots per plant at anthesis stage; NRA_BW_, nodal roots per unit area before winter; NRA_JS_, nodal roots per unit area at jointing stage; NRA_AS_, nodal roots per unit area at anthesis stage.

**Table 2:** N accumulation in grains, post-anthesis nitrogen absorption, nitrogen translocation, the ratio of nitrogen translocation to N accumulation in grains, and the nitrogen harvest index.

Season	Seed Rate (m^−2^)	GN (kg ha^−1^)		NU_post_ (kg ha^−1^)		NT (kg ha^−1^)		NT/GN (%)		NHI (%)	
		NDS	WBS	NDS	WBS	NDS	WBS	NDS	WBS	NDS	WBS
2020−	100	137.8	146.3	70.6	72.1	67.2	74.2	48.8	50.7	62.2	64.3 #
2021	200	170.7	184.3 #	78.3	80.4	92.4	103.9 #	54.1	56.4	65.3	67.4
	300	185.9	204.4 #	83.9	87.0	102.0	117.5 #	54.9	57.5	65.4	67.0
	400	188.7	207.9 #	80.3	81.3	108.4	126.6 #	57.4	60.9 #	65.7	65.9 #
	500	185.8	212.7 #	74.1	73.6	111.7	139.1 #	60.1	65.4 #	65.2	68.4 #
	600	175.5	194.7 #	61.8	61.0	113.7	133.7 #	64.8	68.7 #	64.3	65.6
	700	167.6	182.3 #	54.2	54.5	113.4	127.8 #	67.7	70.1	63.3	63.5
	**Mean**	**173.1**	**190.4** #	**71.9**	**72.8**	**101.3**	**117.5** #	**58.3**	**61.4** #	**64.5**	**66.0** #
	LSD	10.6	11.1	6.2	6.4	13.7	14.4	4.8	5.2	2.0	2.2
	ANOVA										
	SM	**		ns		**		*		*	
	SR	**		**		**		**		*	
	SM × SR	**		ns		**		*		*	
2021−	100	145.5	154.3	83.7	84.6	61.8	69.7	42.5	45.2	59.8	62.2 #
2022	200	172.0	185.5 #	91.9	94.0	80.1	91.5 #	46.6	49.3	62.0	64.8 #
	300	188.7	203.9 #	97.1	99.5	91.6	104.4 #	48.6	51.2	61.9	64.5 #
	400	195.8	217.2 #	97.6	98.7	98.2	118.6 #	50.1	54.6 #	61.2	63.6 #
	500	196.3	223.5 #	89.7	92.1	106.6	131.4 #	54.3	58.8 #	61.1	63.5 #
	600	192.4	213.1 #	77.7	79.0	114.7	134.1 #	59.6	62.9	61.6	62.7
	700	182.0	199.7 #	60.1	59.2	122.0	140.5 #	67.0	70.4	62.3	63.1
	**Mean**	**181.8**	**199.6** #	**85.4**	**86.7**	**96.4**	**112.9** #	**52.7**	**56.1** #	**61.4**	**63.5** #
	LSD	11.3	12.0	6.7	7.3	14.1	14.7	5.2	5.5	1.9	2.1
	ANOVA										
	SM	**		ns		**		*		*	
	SR	**		**		**		**		*	
	SM × SR	**		ns		**		*		*	

Mean, the mean value across the seven seed rates under the same sowing method; SM, sowing method; SR, seed rate; GN, grain N accumulation; NU_post_, post-anthesis N uptake; NT, N translocation; NT/GN, the ratio of N translocation to grain N accumulation; NHI, N harvest index. * and **, significant at the 0.05 and 0.01 probability levels, respectively; ns, not significant at the 0.05 probability level. **#** indicates that there is a significant difference between wide-belt sowing (WBS) and narrow-drill sowing (NDS) at the specific seed rate, according to Student’s *t*-test at *α* = 0.05.

**Table 3:** Grain protein concentration, grain protein yield, water absorption rate, wet gluten concentration, and dough stability time with narrow drill sowing (NDS) and wide-belt sowing (WBS) in the 2020–2021 and 2021–2022 growing seasons.

Season	Seed Rate (m^−2^)	GPC (%)		GPY (kg ha^−1^)		WAR (%)		WGC (%)		DST (min)	
		NDS	WBS	NDS	WBS	NDS	WBS	NDS	WBS	NDS	WBS
2020−	100	12.6	12.7	785	834 **#**	48.5	48.6	29.6	29.5	1.39	1.40
2021	200	13.1	13.3	973	1050 **#**	49.0	49.4	30.1	30.3	1.57	1.58
	300	13.4	13.6	1060	1165 **#**	49.4	49.9	30.5	30.8	1.71	1.74
	400	13.7	13.6	1076	1185 **#**	49.7	50.4	31.0	30.8	1.80	1.82
	500	14.2	14.1	1059	1213 **#**	50.5	50.5	31.7	31.9	1.89	1.89
	600	14.9	14.7	1000	1110 **#**	52.1	52.2	33.0	32.6	2.12	2.11
	700	15.5	15.3	955	1039 **#**	53.0	53.2	34.0	34.2	2.21	2.22
	**Mean**	**13.9**	**13.9**	**987**	**1085 #**	**50.3**	**50.6**	**31.4**	**31.4**	**1.81**	**1.82**
	LSD	0.5	0.4	77	81	1.2	1.3	1.1	1.3	0.23	0.24
	ANOVA										
	SM	ns		**		ns		ns		ns	
	SR	**		**		**		**		**	
	SM × SR	ns		**		ns		ns		ns	
2021−	100	11.9	12.1	829	880 **#**	46.4	46.6	28.7	28.5	1.35	1.36
2022	200	12.4	12.6	981	1057 **#**	47.5	47.2	28.9	29.3	1.51	1.52
	300	12.7	12.7	1076	1162 **#**	47.6	48.2	29.6	30.0	1.65	1.67
	400	13.0	13.0	1116	1238 **#**	48.2	48.3	29.7	29.7	1.73	1.74
	500	13.5	13.3	1119	1274 **#**	48.8	48.4	30.5	30.6	1.82	1.81
	600	14.2	14.1	1097	1215 **#**	50.5	50.7	31.7	31.8	2.05	2.03
	700	14.8	14.6	1038	1138 **#**	51.0	50.8	32.7	32.7	2.12	2.13
	**Mean**	**13.2**	**13.2**	**1036**	**1138 #**	**48.6**	**48.6**	**30.3**	**30.4**	**1.75**	**1.75**
	LSD	0.5	0.5	82	90	1.0	1.2	1.2	1.1	0.27	0.24
	ANOVA										
	SM	ns		**		ns		ns		ns	
	SR	**		**		**		**		**	
	SM × SR	ns		**		ns		ns		ns	

Mean, the mean value across the seven seed rates under the same sowing method; SM, sowing method; SR, seed rate; GPC, grain protein concentration; GPY, grain protein yield; WAR, water absorption ratio; WGC, wet gluten content; DST, dough stability time. **, significant at the 0.01 probability level; ns, not significant at the 0.05 probability level. **#** indicates that there is a significant difference between wide-belt sowing (WBS) and narrow-drill sowing (NDS) at the specific seed rate, according to Student’s *t*-test at *α* = 0.05.

## Data Availability

Data are contained within this article.
